# Response to “A False Dichotomy: RCTs and Their Contributions to Evidence-Based Public Health”

**DOI:** 10.9745/GHSP-D-15-00045

**Published:** 2015-03-02

**Authors:** James D Shelton

**Affiliations:** a Editor-in-Chief, Global Health, Science and Practice.

## Abstract

While randomized controlled trials (RCTs) can and do make valuable contributions, they also have severe limitations, including in answering the basic question of “Does it work?” and, even more so, in steering how to proceed with complex public health programming at scale. They deserve no exalted position in the pantheon of methodologies for evidence-based public health.

***See related articles by*** ***Hatt*** ***and*** ***Shelton*****.**

I appreciate the thoughtful response by Hatt et al.^1^ to my editorial on evidence-based public health^2^ and am happy we agree on several points:

1. There is some definite value to randomized trials.
2. Because public health operates in complex program environments, it is generally necessary to lay out a valid “theory of change” or causal pathway to understand and evaluate the intended and actual program effects.
3. Mixed-method approaches are essential to understand what is going on in such complex arenas.

**“Does it work” is always affected by context.** The paradigm Hatt et al. put forward asserts that one strength of randomized trials is to answer definitively, “Does it work?” But for the kinds of complex programs public health must muster, there is generally no absolute answer to that question or to its companion question, “How well does it work?” Rather, the answer much depends on how and in what situation “it” is done. That is often true even for fairly consistent biologic phenomena, such as the wide variation in polio vaccine efficacy that I cited in my editorial. Perhaps the most recent example is the use of antenatal corticosteroids to prevent newborn mortality, which is effective in developed countries with sophisticated health resources but was found to actually *increase* mortality in certain resource-constrained environments.^3^ Yes, randomized trials and other research methods can answer, “Can it work?” and are often fairly generalizable for discrete biologic questions. But for programming at scale, that takes us only part of the way.

**How it might be made to work practicably at scale is the key question for public health.** A good example is the paper by Curry et al. in this issue of GHSP, describing the many programmatic elements implemented to provide contraception very successfully and at fairly large scale in crisis-affected situations.^4^ Had the program failed to assure an effective supply chain, provide competency-based training, ensure good supervision, and mobilize communities, would the results have been so successful? Highly doubtful. And the richness of their evidence is enhanced by their description of the *effect of variation in country context*; for example, policy differences on the availability of contraceptive implants and the impact of poor compensation of health workers in certain places made big differences.

Variation in country context can make big differences in the effects of interventions.

Ironically, some of the randomized studies Hatt et al. cite neither completely answer the question of “Does it work?” nor provide enough understanding through mixed methods of what is going on.

- The study on pay for performance in Rwanda found an increase in such outcomes as institutional deliveries and children's preventive health visits but no increase in completion of 4 prenatal care visits or of full immunization schedules.^5^ And other than some “anecdotal evidence,” we are left wondering about the crucial question of how the incentive system actually may have influenced the behavior of providers and clients.
- The deworming study in Kenya found a decrease in worms and an increase in school participation and attendance.^6^ But there was no discernible impact on anemia (a commonly hypothesized mechanism to affect school attendance) through which deworming might improve school attendance and there was no impact on actual school achievement. Also the study was carried out in a situation with high worm infestation. Might the results be different in areas with less infestation? And we have no direct information from families themselves on what may have influenced school attendance.

Community deworming is actually a prime example of the very kind of variability that undermines the generalizability of randomized trials. Notably, a 2012 Cochrane review of numerous studies on community deworming concluded^7^:

*For haemaglobin and cognition, community deworming seems to have* ***little or no effect****, and the evidence in relation to school attendance and school performance is generally poor, with* ***no obvious or consistent effect****. Our interpretation of these data is that it is probably* ***misleading to justify contemporary deworming programmes*** *[emphasis added] based on consistent benefit on nutrition, haemoglobin, school attendance or school performance as there is simply insufficient reliable information to know whether this is so.*

Most studies failed to show impact, inescapably because situations varied. So actually, the issue of whether an intervention “is effective on a larger scale” has no single answer. And the recent decision in India to extend mass deworming to large populations has been justifiably roundly criticized.^8^

While I really do appreciate randomized studies, perhaps my biggest concern is the “hierarchy” whereby some colleagues place controlled trials at the top of a pyramid as manifestly the best evidence. For understanding public health programming, I see that as quite misguided. Randomized studies help us to understand some things, but they are only one piece of the picture in “triangulating” evidence for programming. And evidence from real-world programming is especially key.

RCTs are only one piece of the picture in triangulating evidence for public health programming.

Building on the core randomized component and adding other methodologies to the conduct of those randomized trials makes them much more useful. The large Mexico PROGRESA conditional cash transfer program focusing on health, education, and nutrition^9^ cited by Hatt et al. is to some extent an example of such a mixed-method approach and was conducted at large scale. My fear, however, is that the desire to control the research environment, and the resulting narrow focus and often artificiality of trials, limits understanding of the potential programmatic practicability. It also may limit the ability to use additional methodologies to help answer whether the intervention might work practicably at scale.

Thus, to answer the questions well of what, how, and why an intervention may have worked, we need lots of methodologies. Ultimately, some of the “best evidence” or gold standard comes from programs already operating successfully at scale, as illustrated by the paper by Curry.^4^ For public health programming, there is no absolute methodological hierarchy. We need to respect and use all legitimate methodologies.
